# Trauma and Hyperthermia

**DOI:** 10.21980/J8.52308

**Published:** 2025-10-31

**Authors:** William Webster, Dallas Beaird, Linda L Herman

**Affiliations:** * Sutter Roseville Medical Center, Department of Emergency Medicine, Roseville, CA; ^ Vituity Healthcare and Medical Staffing Services, St Agnes Medical Center, Department of Emergency Medicine, Fresno, CA

## Abstract

**Audience:**

Emergency medicine residents and medical students on emergency medicine rotation

**Introduction:**

Participating in strenuous activities during hot, humid weather places a person at risk for heat emergencies such as heat exhaustion and heat stroke. When the environmental temperature is greater than 35° Celsius (95◦ F), the body can no longer radiate heat to the environment. When the humidity is greater than 75%, the body can no longer use evaporation for cooling.^1^ The hot, humid environment has effectively eliminated both mechanisms to decrease body heat, and the individual is at risk for exertional heat injury.^1^ Altered mental status that occurs during heat stroke interferes with the ability to obtain an adequate history as to the etiology of hyperthermia and altered mental status. It is imperative that the patient receive cooling measures immediately and that supportive measures be instituted while evaluating the patient for other etiologies including trauma-related injuries.^1^, ^2^, ^3^

**Educational Objectives:**

By the end of this oral board session, examinees will be able to: 1) construct a differential to evaluate a patient with undifferentiated altered mental status and trauma, 2) recognize the signs and symptoms of heat stroke, 3) complete an evaluation of a patient with both hyperthermia and trauma, and 4) demonstrate efficient and correct treatment of a patient with hyperthermia.

**Educational Methods:**

This oral board case followed the standard American Board of Emergency Medicine style case in a tertiary care hospital with access to all specialists and resources needed. This case was tested using seven first-year and five second-year residents in an ACGME (Accreditation Council for Graduate Medical Education) accredited emergency medicine residency program.

**Research Methods:**

Immediate feedback was solicited both from the learners and from the evaluators following the debriefing session. Residents were asked to evaluate the educational value of the case using a 1–5 Likert scale (5 being excellent). Evaluators were asked to score the residents using the ACGME core competencies with a scale of 1–8, 1–4 being unacceptable and 5–8 being acceptable.

**Results:**

Twelve residents (seven PGY-1 and five PGY-2) completed this oral board case. The average score was 5.34/8. Five residents missed zero critical actions. Six residents failed to explore other etiologies of altered mental status in the case once the temperature was requested and given. Three residents did not complete the primary and secondary survey searching for all injuries. Only two residents did not begin appropriate cooling measures.

The learners rated the educational value of the case as 4.8/5. Seven residents reported that the case increased their medical knowledge; five residents reported that it somewhat increased their medical knowledge. All residents rated the case as helpful in preparing to manage this medical condition.

**Discussion:**

The educational content from this case was effective. This is a high acuity moderate occurrence case that requires an extensive differential with a thorough evaluation as well as the recognition for immediate treatment of hyperthermia. This makes this case excellent for practice and discussion for the evaluation of patients with trauma and the treatment of hyperthermia. We learned during implementation that this case has a moderate degree of difficulty but does provide practice in the area of completing a thorough primary and secondary survey and education concerning best practices for treating heat emergencies.

**Topics:**

Altered mental status, heat emergency, heat stroke, hyperthermia, trauma.

## USER GUIDE

**Table:** 

List of Resources:	
Abstract	31
User Guide	33
For Examiner Only	35
Oral Boards Assessment	43
Stimulus	46
Debriefing and Evaluation Pearls	68

**Learner Audience:**

Medical students, interns, junior residents, senior residents

**Time Required for Implementation:**

Case: 15 minutes

Debriefing: 10 minutes

**Recommended number of learners per instructor:**

one to three learners per instructor

**Topics:**

Myopericarditis, pulmonary edema, cardiogenic shock, sepsis, septic shock.

**Objectives:**

By the end of this oral board session, examinees will be able to:

1. Construct a differential to evaluate a patient with undifferentiated altered mental status and trauma
2. Recognize the signs and symptoms of heat stroke
3. Complete an evaluation of a patient with both hyperthermia and trauma
4. Demonstrate efficient and correct treatment of a patient with hyperthermia

### Linked objectives, methods and results

The case was written in oral board format because it was an appropriate topic to provide experience in the oral board format. The case provides an undifferentiated patient associated with trauma. The learner must collect thorough data about the patient by completing a history and physical which includes voicing the primary and secondary survey. The instructor provides the data from the case and the learner forms a differential (Objective 1). The learner then has to determine what diagnostic evaluation should occur (Objective 3). While waiting for the diagnostic evaluation, the learner should recognize the characteristics of heat stroke. If the learner does not, prompts are provided (Objective 2). The learner should begin treatment for the hyperthermia which can be performed in the emergency department (Objective 4).

### Recommended pre-reading for instructor

- LoVecchio F. Heat emergencies. In: Tintinalli JE, Ma O, Yealy DM, et al, eds. *Tintinalli’s Emergency Medicine: A Comprehensive Study Guide, 9*^*th*^ *ed*. McGraw-Hill Education. 2020:1345–1350.
- American College of Surgeons. Appendix B: Hypothermia and heat emergencies. In: Stewart RM, Merrick C, Peterson N, et al, eds. *ATLS*^*®*^ *Advanced Trauma Life Support Student Course Manual*, 10^th^ ed. American College of Surgeons. 2018:265–272.

### Results and tips for successful implementation

This case was initially presented as an oral board exercise in a small group of 12 emergency medicine residents. We organized a schedule that allowed each resident 30 minutes with a proctor: 15 minutes to complete the case, 10 minutes for debriefing, and 5 minutes for scoring. Feedback from the residents was overwhelmingly positive; they found the case both beneficial and realistic. The case proved moderately challenging for both novice and advanced learners, with average scores of 5.25 and 5.45, respectively. We recommend using this case for learners at all levels. Its strengths include providing practice in conducting a thorough history and physical exam within an oral board format, generating a broad differential for altered mental status, interpreting workups, and ordering appropriate investigations. The case also reinforces best practices in managing hyperthermia. Notably, during implementation, all but two residents correctly initiated cooling measures.

While ideally delivered as an oral board scenario, this case can also be adapted for simulation or small group discussions. Regardless of format, it is essential to debrief with learners afterward or supply post-case reading materials to reinforce key concepts.

### Pearls

- Heat stroke is a life-threatening emergency and can be fatal if unrecognized and untreated. The most important clinical features are hyperthermia (>40 °C [> 104°F]) and altered mental status.^1^,^4^
- The body has multiple ways of dissipating heat which include:
  ○ Radiation – transfer of heat waves from a warmer object to a colder object
  ○ Conduction – heat transfer between two objects in direct contact
  ○ Convection – transfer of heat by currents of air or liquid moving across an object
  ○ Evaporation – heat dissipation by vaporization of liquid 4
- The body’s ability to dissipate heat is impaired in a hot, humid environment and may result in heat stroke when combined with exertional heat production during athletic activity.^1^, ^4^
- Multiple methods of cooling are available for treatment of hyperthermia.
  ○ Evaporative method – remove most clothing from the patient, spray with water and apply moving air across the patient to allow for vaporization^1^,^4^,^5^
  ○ Apply ice packs to axillae, groin, and neck which does assist in cooling but should be used with other methods^1^, ^5^
  ○ Cooling blanket – if used, should be placed under the patient so that it does not interfere with evaporative cooling^1^, ^4^
  ○ Cold water lavage of the stomach, urinary bladder, rectum, or peritoneal cavity but is labor intensive and has no evidence of efficacy^1^,^4^,^5^
  ○ Cold water immersion but monitoring of vital signs and cardiac arrhythmias becomes difficult and the method interferes with other treatments^1^,^2^, ^4^, ^5^
  ○ Cardiopulmonary bypass which is effective but invasive and at times difficult to obtain^5^
- Assessment of the trauma patient should be systematic and thorough performing a primary and secondary survey to find all injuries.^6^
- Failure to respond to simple questions may suggest abnormalities in an airway, breathing, circulation, or disability. 6
- Inability to obtain a detailed history should not keep the emergency medicine physician from evaluating or managing a trauma patient. 7

## Figures and Tables

**Table:** 

**Vitals:**	HR 124	BP 168/96	RR 24	Temp 41.1° C (106° C)	SPO2 96% (room air)

**Table:** 

	Milestone	Did not achieve level 1	Level 1	Level 2	Level 3
**1**	**Emergency Stabilization (PC1)**	□ **Did not achieve Level 1**	□ **Recognizes abnormal vital signs**	□ **Recognizes an unstable patient, requiring intervention** **Performs primary assessment** **Discerns data to formulate a diagnostic impression/plan**	□ **Manages and prioritizes critical actions in a critically ill patient** **Reassesses after implementing a stabilizing intervention**
**2**	**Performance of focused history and physical (PC2)**	□ **Did not achieve Level 1**	□ **Performs a reliable, comprehensive history and physical exam**	□ **Performs and communicates a focused history and physical exam based on chief complaint and urgent issues**	□ **Prioritizes essential components of history and physical exam given dynamic circumstances**
**3**	**Diagnostic studies (PC3)**	□ **Did not achieve Level 1**	□ **Determines the necessity of diagnostic studies**	□ **Orders appropriate diagnostic studies** **Performs appropriate bedside diagnostic studies/procedures**	□ **Prioritizes essential testing** **Interprets results of diagnostic studies** **Considers risks, benefits, contraindications, and alternatives to a diagnostic study or procedure**
**4**	**Diagnosis (PC4)**	□ **Did not achieve Level 1**	□ **Considers a list of potential diagnoses**	□ **Considers an appropriate list of potential diagnosis** **May or may not make correct diagnosis**	□ **Makes the appropriate diagnosis** **Considers other potential diagnoses, avoiding premature closure**
**5**	**Pharmacotherapy (PC5)**	□ **Did not achieve Level 1**	□ **Asks patient for drug allergies**	□ **Selects an appropriate medication for therapeutic intervention, considering potential adverse effects**	□ **Selects the most appropriate medication(s) and understands mechanism of action, effect, and potential side effects** **Considers and recognizes drug-drug interactions**
**6**	**Observation and reassessment (PC6)**	□ **Did not achieve Level 1**	□ **Reevaluates patient at least one time during the case**	□ **Reevaluates patient after most therapeutic interventions**	□ **Consistently evaluates the effectiveness of therapies at appropriate intervals**
**7**	**Disposition (PC7)**	□ **Did not achieve Level 1**	□ **Appropriately selects whether to admit or discharge the patient**	□ **Appropriately selects whether to admit or discharge** **Involves the expertise of some of the appropriate specialists**	□ **Educates the patient appropriately about their disposition** **Assigns patient to an appropriate level of care (ICU/Tele/Floor)** **Involves expertise of all appropriate specialists**
**22**	**Patient centered communication (ICS1)**	□ **Did not achieve level 1**	□ **Establishes rapport and demonstrates empathy to patient (and family)** **Listens effectively**	□ **Elicits patient’s reason for seeking health care**	□ **Manages patient expectations in a manner that minimizes potential for stress, conflict, and misunderstanding.**
**23**	**Team management (ICS2)**	□ **Did not achieve level 1**	□ **Recognizes other members of the patient care team during case (nurse, techs)**	□ **Communicates pertinent information to other healthcare colleagues**	□ **Communicates a clear, succinct, and appropriate handoff with specialists and other colleagues** **Communicates effectively with ancillary staff**

**Table:** 

**#1**	**Patient Information Form**
**#2**	**Point-of-care glucose**
**#3**	**Rhythm strip**
**#4**	**12-lead EKG**
**#5**	**Complete blood count**
**#6**	**Basic metabolic panel**
**#7**	**Venous blood gas and lactate**
**#8**	**Type and screen**
**#9**	**Urinalysis**
**#10**	**Seum and urine toxicology**
**#11**	**Liver enzymes, prothrombin time (PT), INR, partial thromboplastin time (PTT)**
**#12**	**Total creatine kinase and Thyroid panel**
**#13**	**Cervical spine CT results**
**#14**	**Chest, Abdomen, Pelvis CT results**
**#15**	**Chest radiograph**
**#16**	**Pelvis radiograph**
**#17**	**Noncontrast CT brain**
**#18**	**Point-of-care ultrasound – FAST**

**Table:** 

**Patient’s Name:**	**J. Doe**
**Age:**	**28-years-old**
**Gender:**	**Male**
**Chief Complaint:**	**altered mental status, possible head trauma**
**Person Providing History:**	**EMS, paramedics**
**Vital Signs:**	
**Temperature:**	**41.1° C (106° F) (Rectal temperature)**
**Blood Pressure:**	**168/96 mmHg**
**Heart Rate:**	**124 beats per minute**
**Respiratory Rate:**	**24 breaths per minute**
**Pulse Oximetry:**	**97% on room air**
**Weight:**	**83.4 kg (183.9 lbs.)**
**Height:**	**70 inches**

**Table:** 

Glucose	122 mg/dL

**Figure:**
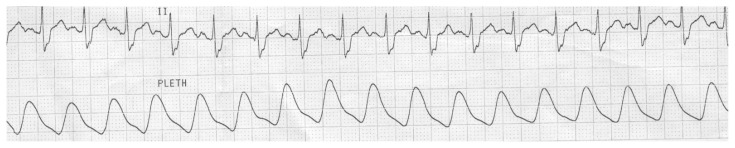
Image Source: Author’s own image.

**Table:** 

Heart rate	125 beats per minute	RR interval	480 msec
P duration	80 msec	QRS duration	80 msec
PR interval	120 msec	QT interval	320 msec
QTc	462 msec		
QT dispersion			
P-Axis	70		
QRS-Axis	38		
T-Axis	68		

**Figure:**
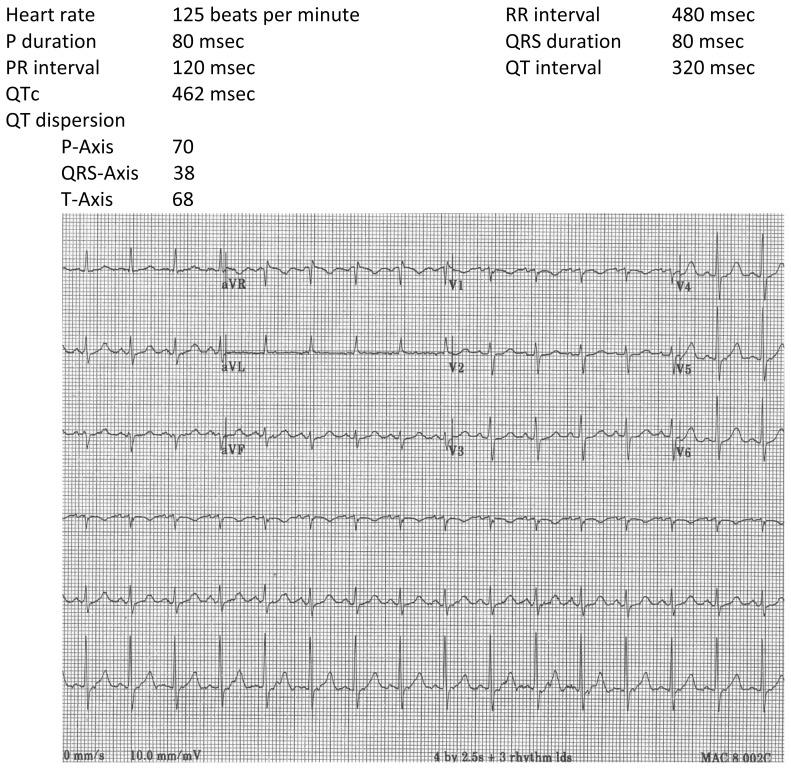
Image Source: Author’s own image.

**Table:** 

WBC	14.7 x 10^3^/μL
Hgb	13.2 g/dL
Hct	39.6%
Platelets	275 x 10^3^/μL
Differential	
Neutrophils	72%
Bands	0%
Lymphocytes	24%
Monocytes	3%
Eosinophils	1%

**Table:** 

Sodium	147 mEq/L
Potassium	4.3 mEq/L
Chloride	106 mEq/L
Bicarbonate	22 mEq/L
Blood Urea Nitrogen (BUN)	22 mg/dL
Creatinine (Cr)	0.6 mg/dL
Glucose	124 mg/dL

**Table:** 

pH	7.4
pCO2	46 mmHg
pO2	52 mmHg
HCO3	25 mEq/L
O2 saturation	98&
Lactate	4.0 mEq/L

**Table:** 

Blood type	A positive

**Table:** 

Appearance	Clear
Color	Yellow
Glucose	Negative
Ketones	1+
Specific Gravity	1.036
Blood	Negative
pH	6.0
Protein	Negative
Nitrite	Negative
Leukocyte	Negative
WBC	0/HPF
RBC	2–3/HPF
Squamous	Cells 0
Bacteria	Negative

**Table:** 

Alcohol	Negative

**Table:** 

Cocaine	Negative
Cannabinoid	Negative
Phencyclidine	Negative
Amphetamine/Methamphetamine	Negative
Opiates	Negative
Benzodiazepine	Negative
Barbituates	Negative
Fentanyl	Negative

**Table:** 

Albumin	3.7 g/dL
Bilirubin, Total	1.0 mg/dL
Bilirubin, Direct	0.3 mg/dL
Alkaline Phosphatase	116 units/L
AST/GOT	28 units/L
ALT/GPT	25 units/L
Total Protein	7.1 gm/dL

**Table:** 

PT	10.7 seconds
INR	.91
PTT	28.1 seconds

**Table:** 

Creatine Kinase	980 units/L

**Table:** 

Thyroid Stimulating Hormone (TSH)	2.45 uIU/mL
Free thyroxine (Free T4)	1.02 ng/dL
Free Triiodothyronine (Free T3)	2.7 pg/mL

**Figure:**
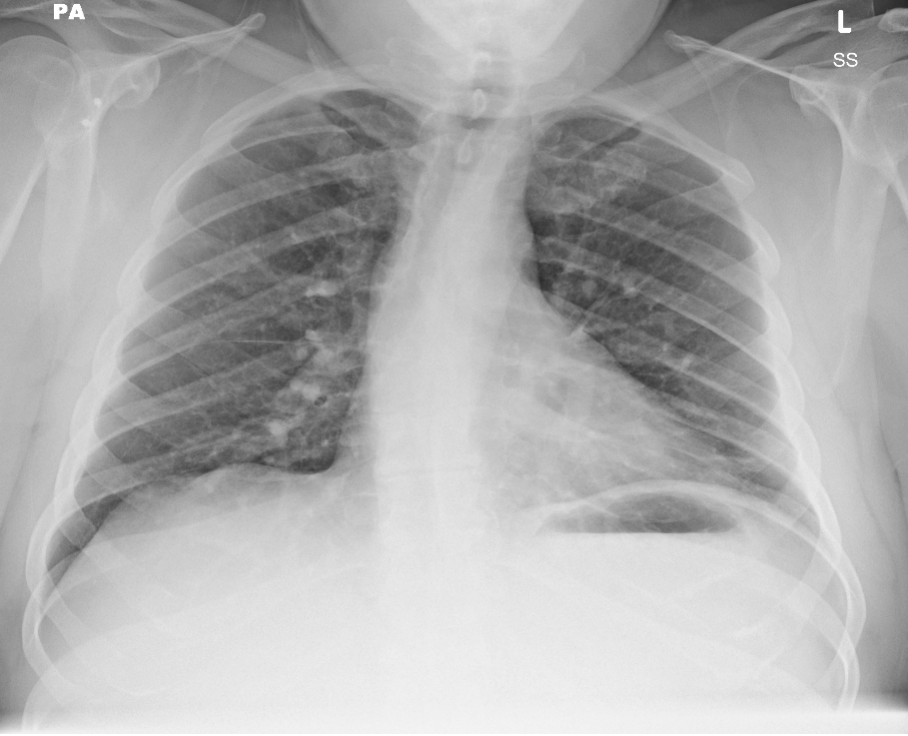
Image Source: Author’s own image.

**Figure:**
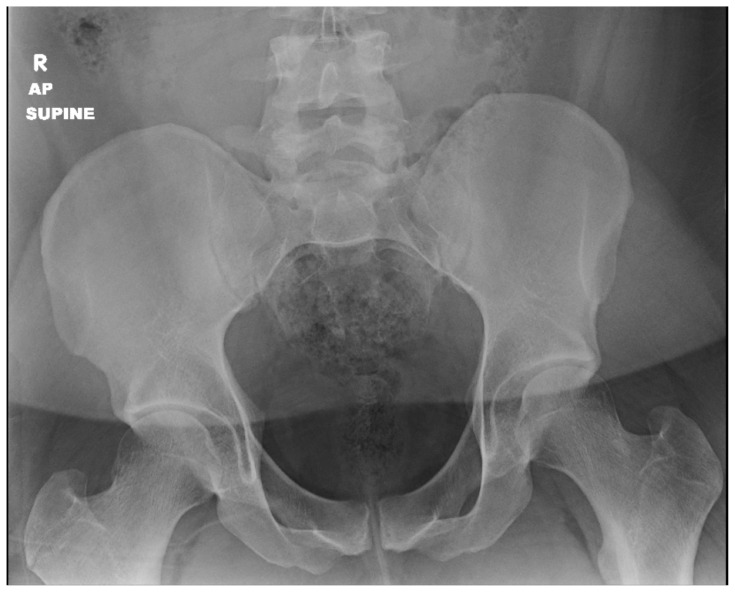
Image Source: Author’s own image.

**Figure:**
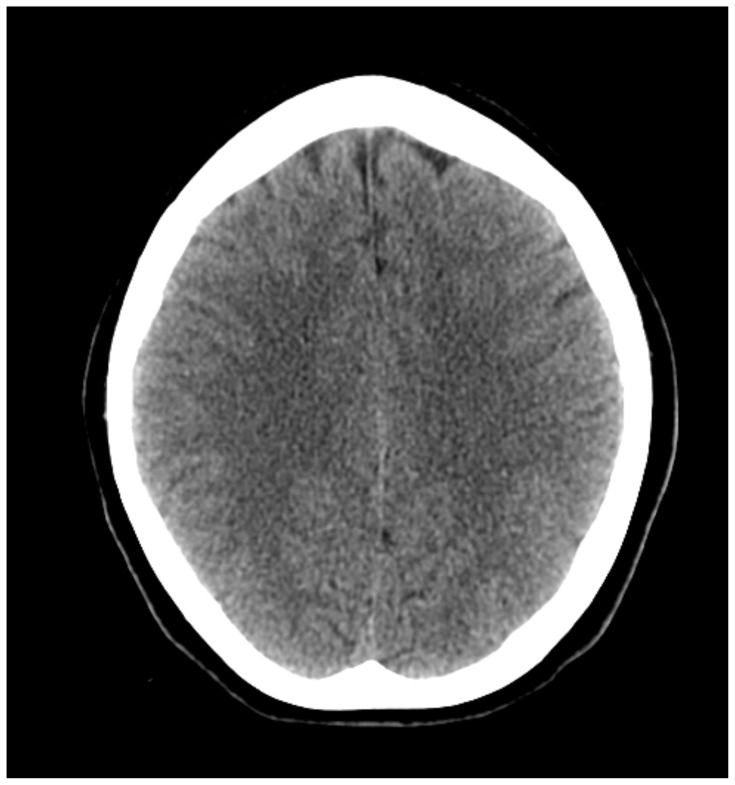
Image Source: Author’s own image.

**Figure:**
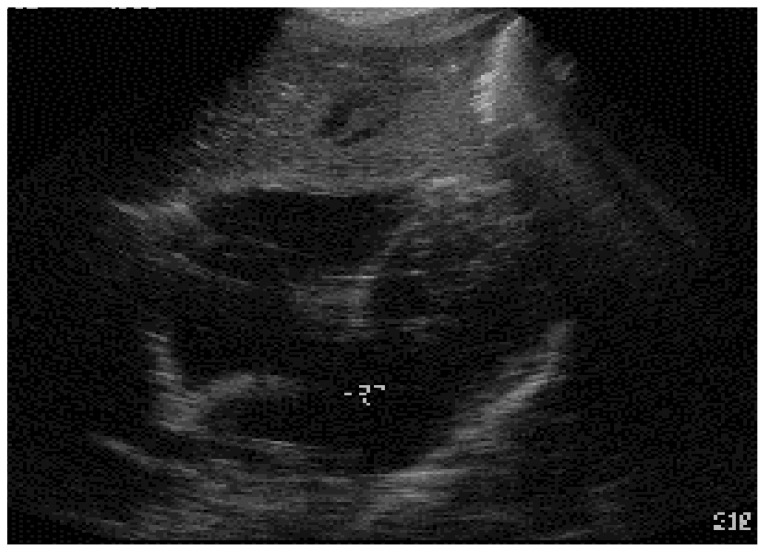
Image Source: Author’s own image.

**Figure:**
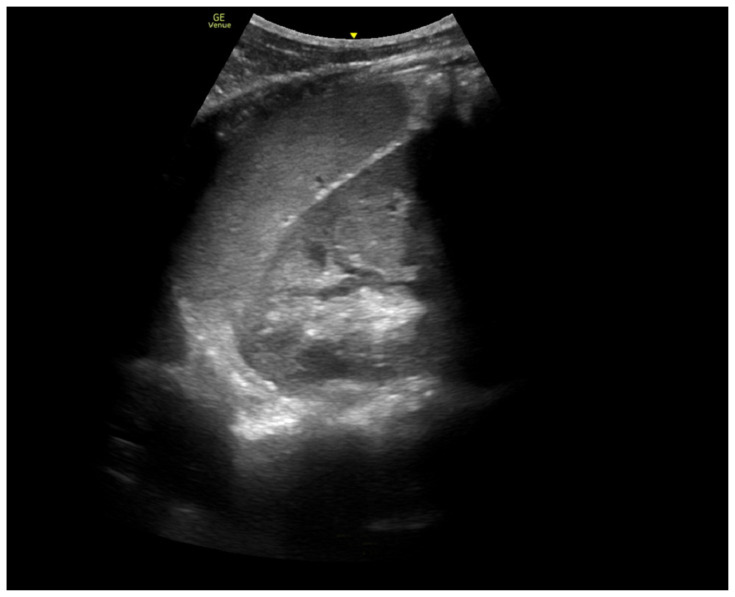
Image Source: Author’s own image.

**Figure:**
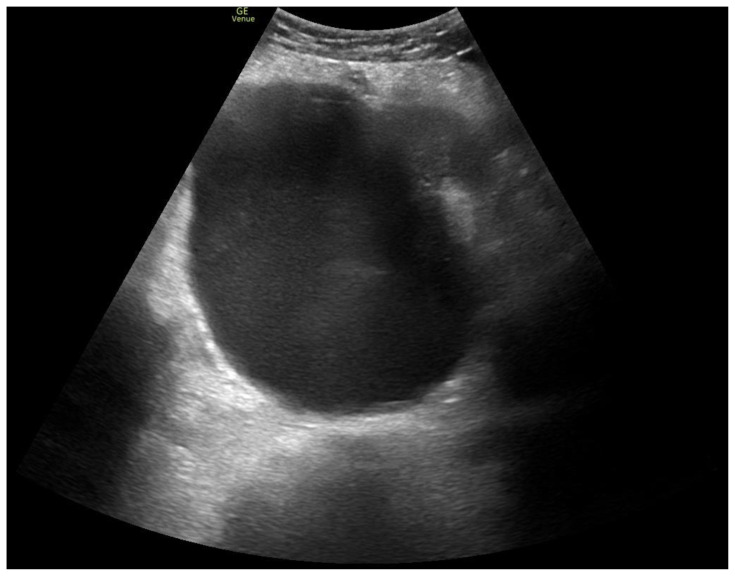
Image Source: Author’s own image.

**Figure:**
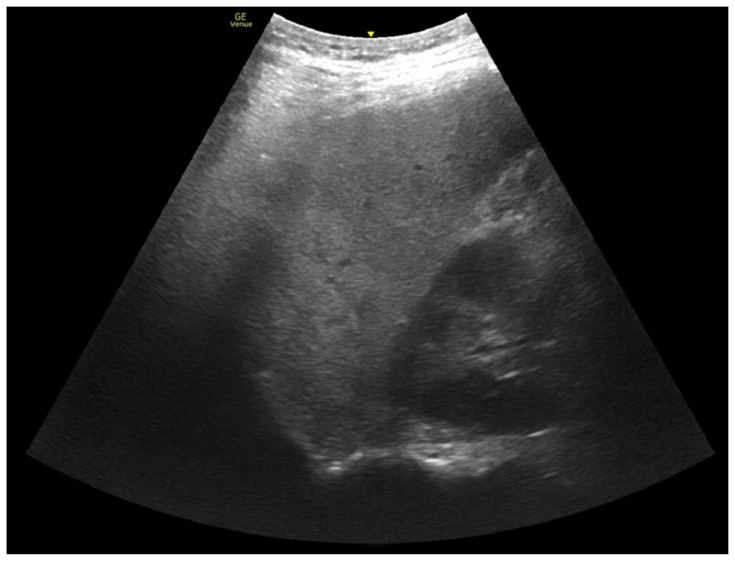
Image Source: Author’s own image.

## FOR EXAMINER ONLY

### Oral Case Summary

**Diagnosis: Trauma and Hyperthermia**

**Case Summary:** The patient is a 28-year-old man, Mr. D, who is brought from a bike path by the paramedics after being found by bystanders lying on the ground next to his bike. The ambient temperature is 102° F with 80% humidity. A bicyclist called 911 and reported the incident. No one at the scene knows the patient. The patient appears to have head trauma even though he was wearing a helmet which was noted by medics to be intact with some scoff marks. The paramedics removed the helmet and applied a backboard and cervical collar. When the patient was placed in the ambulance, he began to thrash about and then pulled out his IV. He was physically restrained but another IV was not inserted. Vital signs: BP-154/92, Pulse-120, Respiratory Rate-20. No history is obtainable from the patient.

**Order of Case:** The patient arrives in the ED lying on a backboard and a cervical collar. He is lying with his eyes closed, pulling at the restraints. He does not react to any verbal communication, does not follow commands, and cannot give a medical history. He does have identification in a pouch around his waist.

The candidate obtains a brief history from the paramedics. The paramedics should offer the information about the ambient temperature at this time. The learner performs a primary survey. The patient’s airway is intact, the patient can clench his teeth, appears to swallow, and has a gag. The patient’s respiration is intact, the patient is breathing, the chest moves equally with good air exchange, and breath sounds are clear with no wounds on the chest. The patient’s circulation is intact, and the learner finds that peripheral pulses are strong and bounding, capillary refill is less than two seconds, and there are no wounds with active bleeding. At this time, the proctor discloses to the learner that the patient feels warm. The learner should request a rectal temperature at this time. The patient’s disability is assessed and can move all extremities spontaneously but does not follow commands. The learner requests that the patient be completely undressed, two IVs be started, the patient be placed on the monitor and pulse oximeter, obtain a room air oximeter reading, and start the patient on a nonrebreather mask at 15 liters of oxygen.

The learner requests a point-of-care-glucose and then requests the nurse draw blood. If the learner has not realized that a temperature has not been reported, the proctor should act as the nurse, and after the IVs are started, make comments about the patient’s feeling warm. The learner requests that the nursing staff look for ID and attempt to notify the family. The learner reads the monitor strip. The learner considers the differential for hyperthermia with trauma and orders CXR, CT brain and cervical spine without contrast, and CT chest, abdomen, and pelvis with contrast because traumatic injuries cannot be ruled out clinically due to the patient’s altered mental status. The learner also orders a complete blood count (CBC), metabolic panel (BMP), liver function tests (LFTs), prothrombin time (PT), INR, partial thromboplastin time (PTT), total creatine kinase, thyroid panel, Type and Screen, alcohol level, blood cultures, urinalysis, urine toxicology, and electrocardiogram. The learner begins treatment for hyperthermia by having the nurse place ice packs at the groin and axillae. The learner asks the nurse to move the large ED fan into the room and then spray the patient with water mist. The learner asks the nurse to repeat this each time the patient dries due to the fan until the rectal temperature is decreased to 40°C (104° F). Cool IV fluids are ordered by placing the IV tubing in ice water.

The learner may elect to perform other methods of cooling although not required. If the learner wants to perform gastric lavage, the patient must be intubated first. Also, cooling with peritoneal lavage is allowed, but the learner must place the catheter. The learner may also decide to give acetaminophen per rectum or intravenously although antipyretics are not effective in heat stroke. The candidate may ask for a cooling blanket; if used, it should be placed under the patient, and the evaporative method should be continued. The learner may order irrigation of the bladder with cold liquid.

The learner performs a FAST exam and interprets the results as negative. Next, a secondary survey is performed, followed by a GU exam and rectal which must be performed before the insertion of the foley. The CXR Is provided and interpreted.

Even though the heart rate is rapid, the blood pressure is adequate, so the learner should hold IV fluids until an intracranial traumatic injury is ruled out. Repeat vital signs: BP 160/92, HR 116, RR 20. The learner orders a sedative so that the patient can be transported to CT for advanced imaging. Numerous sedatives can be chosen which include any benzodiazepine or ketamine.^8^ Haloperidol should not be used because of the presence of hyperthermia. When the patient returns from CT, the learner interprets the CT brain and begins fluid at a rate of 150–300 ml/hour. The learner reviews all lab results and advanced imaging results. The learner continues to evaluate the possible differential, orders Rocephin and vancomycin, and orders sedation to perform a lumbar puncture.

The wife arrives and a past medical history is obtained. The patient is an avid cyclist and never fails to ride every day no matter the weather. The patient does not smoke, uses alcohol rarely, does not use illicit drugs, has no significant medical history, and only takes vitamins every day. The learner explains everything to the wife and allows the wife to see the patient. The nurse reports that the temperature is now 39.4°C (103° F) and that the patient is more cooperative but still confused.

The learner gives report to the intensivist and admits the patient.

**Disposition:** Admission to the Intensive Care Unit (ICU)

**Critical Actions:**

1. Orders point-of-care glucose. (data acquisition, system-based practice)
2. Requests rectal temperature be performed. (data acquisition)
3. Begin appropriate cooling methods. (problem-solving, patient care, practice-based learning)
4. Performs genital and rectal exams before ordering the indwelling urinary catheter. (data acquisition, patient care)
5. Admits patient to ICU. (patient care, interpersonal skills)

### Historical Information

**Chief Complaint:** Possible Trauma patient from bicycle accident

**History of Present Illness:** The patient is a 28-year-old man, with unknown medical history, brought by the paramedics. The paramedics state that they were called to the bike path on the forest preserve by a bicyclist with a cell phone reporting the male who was found unresponsive next to a bicycle. The other bicyclist does not know the victim. When paramedics arrived on the scene, the patient was lying on the bike path unresponsive. He did have a bicycle helmet. They cut the straps and removed it before placing the patient on a backboard and cervical collar. As soon as they got the patient into the ambulance, he began to thrash about and pulled his IV out. They had to restrain him, and they were unable to initiate another IV. Vital signs in the field were BP 154/92, HR 120, RR 20. The patient has continued to thrash about, does not follow commands, and appears not to understand anything the paramedics have said to him. They transported him to the emergency department.

**Past Medical History:** unknown

**Past Surgical History:** abdominal scar in the right lower quadrant.

**Patient’s Medications:** unknown

**Allergies:** unknown

**Social history:**

- Smoking: unknown
- Tobacco: unknown
- Drug use: unknown

**Family history:** unknown

### Physical Exam Information

**Weight:** 83.4 kg

**General appearance:** Well-developed, muscular male lying flat on the cart with backboard and cervical collar and all four extremities in soft restraints.

**Primary survey:**

- **Airway:** patent, clenches teeth, appears to swallow, has a gag
- **Breathing:** breath sounds present bilaterally, normal effort
- **Circulation**: bounding pulses peripherally, tachycardic, hot skin, capillary refill less 2 seconds, no active bleeding, no wounds
- **Disability:** moves all extremities spontaneously, opens eyes spontaneously, does not follow commands, and although speaks occasionally does not answer questions

**Physical examination:**

**General appearance:** Well-developed, muscular male lying flat on the cart with backboard and cervical collar, and all four extremities in soft restraints.

- **HEENT:** *If not normal, can give specific information below:*
  ○ **Head:** edema and ecchymosis to left occipital area, no edema or ecchymosis about the face, no battle sign, no raccoon eyes, no step-off deformities
  ○ **Eyes:** within normal limits
  ○ **Ears:** within normal limits
  ○ **Nose:** within normal limits
  ○ **Oropharynx/Throat:** pharynx clear, dry mucous membranes, opens and closes mouth easily, teeth are aligned, when attempting to look at the pharynx patient attempts to clench teeth
- **Neck:** trachea midline, no jugular vein distension (JVD), no evidence of trauma, patient is too altered to evaluate for cervical tenderness
- **Chest:** Breath sounds clear and equal bilaterally, no ecchymosis, no crepitus, equal excursion bilaterally, no response to checking for tenderness
- **Cardiovascular:** Apical regular, normal heart tones, tachycardic, peripheral pulses equal and strong bilaterally
- **Abdominal/GI:** soft, active bowel sounds, no distention, no organomegaly, no response to checking for tenderness
- **Genitourinary:** within normal limits
- **Rectal:** normal tone, guaiac negative, brown stool in the vault, but it is warm in the vault, prostate palpable and normal
- **Extremities:** no clubbing or cyanosis, no deformities, abrasions over both knees and left elbow but the patient is moving all of his extremities spontaneously and appears to have good range of motion
- **Back:** within normal limits, no response to questions concerning tenderness
- **Neuro:** Glasgow Coma Scale – Eye opening – spontaneous = 4, Verbal response – moans or unintelligible sounds = 2, Motor response – movement or withdrawal to pain = 4, Total =10
- **Skin:** hot/dry, no rashes noted, normal turgor
- **Lymph:** within normal limits
- **Psych:** confused, agitated

### Critical Actions and Cueing Guidelines

1. **Critical Action 1: Orders point-of-care glucose. (patient care, system-based practice)**
  This critical action is met by the learner’s requesting that a point-of-care glucose be performed and then interpreting the value to be within normal limits and that hypoglycemia is not the etiology for the altered mental status.
  Cueing Guideline:
  *If the learner does not order a point-of-care glucose, the nurse should ask when drawing the blood if the learner would like to have any tests done immediately. The cueing guideline should not be more specific than that prompt.*
2. **Critical Action 2: Requests rectal temperature. (patient care, medical knowledge)**
  This critical action is met by the learner’s realizing that a temperature has not been provided and requesting a rectal temperature because the patient will not cooperate with an oral temperature.
  Cueing Guideline:
  *When the learner performs the primary survey, the warmth of the skin should be offered when the cardiovascular assessment is performed. If the learner does not request a temperature at this time, the nurse should say at the time the intravenous catheters are inserted, “When I started the IV, the patient’s arms feel really hot.”*
3. **Critical Action 3: Begins appropriate cooling methods. (patient care, medical knowledge)**
  This critical action is met by the learner’s ordering the nurse to place ice packs at the groin and axillae. The learner asks the nurse to move the large ED fan into the room and then spray the patient with a water mist to start evaporative cooling. The learner asks the nurse to repeat this each time the patient dries due to the fan until the rectal temperature is decreased to 40°C (104° F). Cool IV fluids are ordered by placing the IV tubing in ice water.
  Cueing Guideline:
  *If the learner does not begin evaporative cooling with the other measures above, the nurse should tell the learner that the temperature is not decreasing. If the learner still does not begin the above treatment, the nurse should ask, “Are we using the best method to decrease the temperature?”*
4. **Critical Action 4: Performs genital and rectal examination before ordering the indwelling urinary catheter to be placed. (patient care)**
  This critical action is met by the learner’s stating that he will perform a rectal exam and then requests information about the presence of blood at the meatus or in the rectal vault and location of the prostate.
  Cueing Guideline:
  *If the learner does not perform the genital and rectal examination before ordering a foley, the ED technician should ask, “Is it safe to insert the Foley now?”*
5. **Critical Action 5: Admits the patient to the ICU. (patient care, interpersonal skills)**
  This critical action is met by the learner’s requesting to speak to the intensivist on-call and giving a concise and accurate report which includes presentation, evaluation, treatment, and diagnoses.
  Cueing Guideline:
  *If the learner does not decide to admit the patient to the ICU, the nurse should say, “I don’t think that they will accept this patient on the floor because he is still confused and his temperature is still 103 degrees.”*

## ORAL BOARDS ASSESSMENT

*Trauma and Hyperthermia*

Learner: _________________________________________

**Critical Actions:**

□ Orders point-of-care glucose (patient care, system-based practice)
□ Requests rectal temperature be performed. (patient care)
□ Begin appropriate cooling methods. (patient care, medical knowledge)
□ Performs genital and rectal exams before ordering the indwelling urinary catheter. (patient care)
□ Admits patient to intensive care. (patient care, interpersonal skills)

**Summative and formative comments:**

**Milestone assessment:**

### Stimulus Inventory

#### Stimulus #13

##### CT Cervical Spine

No cervical vertebral fracture or malalignment. No prevertebral soft tissue swelling. The atlantooccipital and atlantoaxial joints are congruent.

#### Stimulus #14

##### CT Chest, Abdomen and Pelvis

Lower neck and chest wall: No axillary lymphadenopathy, no subcutaneous emphysema

Mediastinum and Hila: No lymphadenopathy, no evidence of hematomas

Cardiovascular: Adequate opacification of the pulmonary arterial tree, no disruptions of the bronchial tree

Lungs, airways, and pleura: no areas of consolidation, no evidence of pulmonary contusions, lungs fully inflated, no pleural effusions, no pneumothorax, no hemothorax

Osseous structures: no acute fractures of the thoracic or lumbosacral spine, no rib fractures

Liver: Unremarkable

Biliary system: no biliary abnormality identified

Pancreas: unremarkable

Spleen: intact, no hemorrhage, no contusion

Adrenal glands: unremarkable

Kidneys, ureters and bladder: unremarkable

Bowel: no evidence of contusion, no evidence of obstruction

Lymph nodes/mesentery: no enlarged lymph nodes, no evidence of mesenteric hemorrhage

Free fluid/free air: no ascites or free air

Vasculature: unremarkable

Abdominal/pelvic wall: unremarkable

Impression: no acute intrathoracic or intraabdominal pathology.

#### Stimulus #18

##### Point-of-care Ultrasound: FAST

Subxiphoid view

Splenorenal recess

Suprapubic Window

Hepatorenal space

## DEBRIEFING AND EVALUATION PEARLS

### Trauma and Hyperthermia

- Heat stroke is a life-threatening emergency and can be fatal if unrecognized and untreated. The most important clinical features are hyperthermia (>40 ° C [> 104° F]) and altered mental status.^1^,^4^
- The body has multiple ways of dissipating heat which include:
  ○ Radiation – transfer of heat waves from a warmer object to a colder object
  ○ Conduction – heat transfer between two objects in direct contact
  ○ Convection – transfer of heat by currents of air or liquid moving across an object
  ○ Evaporation – heat dissipation by vaporization of liquid 4
- The body’s ability to dissipate heat is impaired in a hot, humid environment and may result in heat stroke when combined with exertional heat production during athletic activity.^1^, ^4^
- Multiple methods of cooling are available for treatment of hyperthermia.
  ○ Evaporative method – remove most clothing from the patient, spray with water and apply moving air across the patient to allow for vaporization^1^,^4^,^5^
  ○ Apply ice packs to axillae, groin, and neck which does assist in cooling but should be used with other methods^1^, ^5^
  ○ Cooling blanket – if used, should be placed under the patient so that it does not interfere with evaporative cooling^1^, ^4^
  ○ Cold water lavage of the stomach, urinary bladder, rectum, or peritoneal cavity but is labor intensive and has no evidence of efficacy^1^,^4^,^5^
  ○ Cold water immersion but monitoring of vital signs and cardiac arrhythmias becomes difficult and the method interferes with other treatments^1^,^2^, ^4^, ^5^
  ○ Cardiopulmonary bypass which is effective but invasive and at times difficult to obtain^5^
- Assessment of the trauma patient should be systematic and thorough performing a primary and secondary survey to find all injuries.^6^
- Failure to respond to simple questions may suggest abnormalities in an airway, breathing, circulation, or disability. 6
- Inability to obtain a detailed history should not keep the emergency medicine physician from evaluating or managing a trauma patient. 7
